# Comment: naming convention for gradient system transfer function and gradient system frequency response for magnetic resonance imaging encoding field characterization

**DOI:** 10.1007/s10334-025-01314-7

**Published:** 2026-01-30

**Authors:** Niklas Wehkamp, Patrick Hucker, Johannes Fischer, Andreas Greiner, Jon-Fredrik Nielsen, Maxim Zaitsev, Robert Dehnert

**Affiliations:** 1 Division of Medical Physics, Department of Diagnostic and Interventional Radiology, University Medical Center Freiburg, Faculty of Medicine, University of Freiburg, Freiburg, Germany; 2 Laboratory for Simulation, Department of Microsystems Engineering (IMTEK), University of Freiburg, Freiburg, Germany; 3 Department of Biomedical Engineering, University of Michigan, Ann Arbor, Michigan USA; 4 Institute of Automatic Control, School of Electrical, Information and Media Engineering, University of Wuppertal, Wuppertal, Germany

**Keywords:** Gradient impulse response function (GIRF), Gradient transfer function (GTF), Gradient system transfer function (GSTF), Gradient frequency response (GFR), Frequency response function (FRF), Gradient modulation transfer function (GMTF), Magnetic resonance imaging (MRI), System characterization

## Abstract

The frequency response and transfer function of a system are closely related, but distinct concepts from a control system theory and signal processing perspective. Unfortunately, these concepts have been used inconsistently in the magnetic resonance imaging (MRI) literature for gradient characterization. This note highlights the differences, with the intention to establish a common naming convention, consistent with other engineering fields. This will facilitate communication between colleagues with a different research background, promoting knowledge transfer and potentially alleviate shortcomings that have resulted from the incorrect usage of the term “transfer function” for gradient characterization in the past.

## Introduction

Spatial encoding in magnetic resonance imaging (MRI) is achieved by the application of time-varying magnetic fields, typically designed for the resonance frequency of the nucleus of interest to vary linearly with the spatial direction upon the activation of the corresponding gradient coil. In conventional MRI systems, three gradient coils are required corresponding to the spatial directions, x, y, z, to achieve three-dimensional encoding. Both spatial and temporal fidelity of these fields are important for the accuracy of the resulting images. In the MRI literature for encoding field characterization, the gradient field response of the gradient system to the requested nominal gradient waveform is a critical metric describing the systems’ behavior. The description assumes that the gradient system, comprising gradient waveform generator, gradient amplifier, gradient coil, conductive components of the cryostat, and the main magnet, behaves as a linear and time-invariant (LTI) system. A system is called linear time-invariant if it satisfies the principles of superposition (linearity) and remains unchanged over time. This means that the system’s response to a weighted sum of inputs is the weighted sum of the responses to each input, and shifting the input in time results in an identically shifted output. The effect of the magnetic field on a spin system is conveniently described by the k-space formalism [1], where the k-vector is defined as a time integral of the gradient vector over the relevant time period. Various MR imaging methods can be characterized by their k-space trajectories. The assumption of an LTI system has been used successfully in MRI to predict systematic k-space trajectory deviations and to reduce resulting image artifacts [2, 3], especially in fast imaging sequences [4]. To correct for the trajectory deviations, approaches have been presented that rely on a characterization of the gradient system to predict trajectory deviations during the image reconstruction [2] or to prospectively apply a pre-emphasis to the gradient waveforms to improve the fidelity of the trajectory actually realized by the gradient system [3].

However, there appears to be a widespread misuse of terminology in the MRI literature, where terms gradient transfer function (GTF) [5], gradient modulation transfer function (GMTF) [6] or gradient system transfer function (GSTF) [7–10] are commonly used to refer to what is, in fact, the frequency response of the system. This frequency response should more accurately be termed the gradient system frequency response (GSFR). The current terminology can be confusing for new colleagues and introduces unnecessary ambiguity around a topic that should, in principle, be straightforward—if standard terminology is used. The first author had previously adopted the incorrect naming convention as well [11]. The confusion is further compounded by the fact that some authors even use the term gradient impulse response function (GIRF) to refer to the frequency response [2, 12]. As laid out in the next section, the GIRF is in the time domain and not the frequency domain. However, in control theory and signal processing, standardized terminology is consistently used to describe LTI systems, such as step response, impulse response, frequency response, and transfer function.

In this note, we aim to clarify the distinctions between transfer function, frequency response, and impulse response, and to discuss their implications for gradient system characterization and gradient pre-emphasis. Establishing consistent naming conventions is important, as it enables researchers to effectively build on solutions from other disciplines, rather than unnecessarily reinventing methods that are already well understood under different names.

## Standard literature definitions

The concepts of the transfer function, frequency response, and impulse response function are central to the analysis and design of linear time-invariant (LTI) systems in both control theory and signal processing. While closely related, these standard texts [13–19] consistently differentiate them by their domain, scope, and primary application.

### The transfer function

The transfer function is recognized as the fundamental mathematical model that characterizes the dynamic input–output relationship of an LTI system. It is defined in a complex frequency domain, typically the Laplace domain with the complex s-plane for continuous-time systems H(s) or the Z-domain for discrete-time systems H(z).

The transfer function H(s) is given by $H\left(s\right)=\frac{Y\left(s\right)}{X\left(s\right)},$ representing the ratio of the Laplace transform of the output signal Y(s) to that of the Laplace transform of the input signal X(s), assuming all initial conditions are zero [13–15].

Similarly, for discrete-time systems, H(z) is defined as the ratio of the Z-transform of the output to the Z-transform of the input [16, 17].

The transfer function has the following properties:

● Comprehensive system characterization: This provides a full algebraic representation of a system’s differential or difference equations. This enables the analysis of all aspects of dynamic systems, including stability, controllability, and observability [18, 19].
● Poles and zeros: The poles of the transfer function, which are the zeros of the denominator polynomial, determine the system’s stability and its natural modes of response. The zeros of the transfer function, which are the roots of the numerator polynomial, influence the transient response and frequency-dependent behavior [13, 14].
● Generality of application: Given an analytical expression for the transfer function, the system’s time-domain response to arbitrary inputs (e.g., impulse, step, ramp) can be derived via inverse Laplace or Z-transform [13].
● Experimental determination (system Identification): When the transfer function is to be determined from experimental data (e.g., from frequency-domain measurements), additional system modeling and parameter estimation are required. Obtaining the transfer function from measured input/output data is a process referred to as parametric system identification [13]. This involves a "curve-fitting problem" where the coefficients of a rational polynomial transfer function are estimated to best match the observed system behavior. This process can leverage either time-domain transient response data or frequency-domain measurements, aiming to derive a compact, analytical model for the system [20].

In short, the transfer function H(s) provides a complete mathematical characterization in the form of a rational polynomial of an LTI system in the complex frequency domain, encoding the input–output behavior, frequency response, and pole-zero structure. An example procedure to determine the transfer function from the frequency response is described in Appendix A.

### The frequency response

The frequency response is considered a specific evaluation of the transfer function that characterizes the behavior of an LTI system when subjected to sinusoidal inputs. As defined in signal processing [16, 17] and control engineering literature [15, 18], the frequency response is obtained by substituting s=jω into the continuous-time transfer function H(s), or $z={e}^{j\omega }$ into the discrete-time transfer function H(z) as follows:

$$H\left(j\omega \right)= H\left(s\right) {|}_{s=j\omega },$$

$$H({e}^{j\omega })=H(z) {|}_{z={e}^{j\omega }.}$$

This procedure essentially ignores the real part of s, focusing solely on the system‘s behavior under steady-state sinusoidal excitation at the real-valued angular frequency ω.

The frequency response has the following properties:

- Steady-state sinusoidal behavior: The most common conceptual understanding is that H(jω) quantifies the system‘s steady-state response to sinusoidal inputs. If a pure sinusoid of frequency ω is applied, the output will also be a sinusoid of the same frequency, after transients have decayed. However, the output amplitude will be scaled by the magnitude $\left|H(j\omega )\right|$ and its phase shifted by $\angle H(j\omega )$. This behavior forms the basis for frequency-domain analysis tools such as Bode plots [13, 14].
- Relation to the impulse response: The frequency response H(jω) can be calculated by the Fourier transform of the system‘s impulse response h(t). Since the impulse response captures all time-domain dynamics (both transient and steady-state), its Fourier transform provides the full frequency-domain characterization.
- Experimental determination via transient methods: The mathematical relationship between the impulse response and frequency response allows for experimental determination of H(jω) without requiring steady-state conditions at each frequency. By applying a broadband, short-duration input (such as a precisely known triangle pulse, a pseudo-random noise-like signal or a chirp pulse) and computing the Fourier transforms of both the input X(jω) and the resulting output Y(jω), the frequency response data $H(j\omega )=Y(j\omega )/X(j\omega )$ can be obtained across a range of frequencies [21]. This approach avoids the need to wait for steady state at each individual frequency [22]. This is the standard method for gradient characterization in MRI as well.
- Visualization: The frequency response is commonly visualized using Bode plots, which display magnitude (in decibels, computed as $20{log}_{10}(\mid H(j\omega )\mid )$) and phase (in degrees) versus logarithmic frequency. In the MRI literature, however, linear frequency plots are often preferred, as they better highlight the low-pass behavior and relevant resonances in the frequency range of approximately 400–3000 Hz.

The frequency response H(jω) is a specific evaluation of the transfer function on the imaginary axis of the complex s-plane. While its conceptual definition ties to steady state, its practical determination can also efficiently leverage the complete information embedded within a transient response through Fourier analysis. The resulting frequency response data can serve as the foundation for the derivation of a parametric transfer function model.

### The impulse response function

The impulse response function is a fundamental time-domain characteristic of an LTI system that defines its intrinsic behavior. It is the system‘s output when the input is an idealized Dirac delta function (for continuous-time systems, h(t)) or a unit sample sequence (for discrete-time systems, h[n]), assuming the system starts at rest [13, 14, 16].

Key aspects of the impulse response include:

- Complete system characterization: A crucial property of LTI systems is that their impulse response uniquely characterizes their behavior. Knowing h(t) allows the system‘s output y(t) to be computed for *any* input x(t) via convolution: y(t)=x(t)∗h(t) [16, 18, 19].
- Link to the frequency domain: The impulse response is also the direct bridge to the frequency domain. The transfer function (H(s) or H(z)) is its Laplace or Z-transform, and the frequency response (H(jω)) is its Fourier transform [15, 17]. In practice, the experimental transient responses (e.g., from a triangle or a box-car pulse) can be used to derive a system‘s frequency response [21, 22].

In essence, the impulse response represents the system’s inherent dynamic fingerprint in the time domain, from which all other properties, including frequency response and transfer function, can be mathematically derived.

## Discussion

When comparing the MRI gradient characterization literature to the previous section, it is obvious that several MRI papers [2, 3, 5–10] refer to the transfer function or impulse response for what is the frequency response in the control theory and engineering literature [13–19]. The current use of terms such as "gradient transfer function (GTF)" or "gradient system transfer function (GSTF)" in the MRI community introduces conceptual and practical disadvantages. A more standard literature coherent name could, for example, be the “gradient system's frequency response (GSFR)”. The current terminology can be confusing for new colleagues and causes unnecessary uncertainty around a topic that generally would not be that complicated at all if the standard terminology were used. Adopting the established nomenclature will not only enhance interdisciplinary communication, but also unlock the analytical advantages of the transfer function.

While the frequency response, H(jω), derived from the gradient impulse response function (GIRF) and subsequent Fourier transform, captures the system‘s dynamic characteristics across frequencies [16], it is fundamentally a projection of the system‘s behavior onto the imaginary axis of the complex frequency domain. For many MRI applications, particularly those focusing on signal fidelity and basic sequence design, the measured frequency response provides valuable information for trajectory correction and pre-emphasis, as currently practiced.

However, limiting the characterization to the frequency response, rather than explicitly deriving and utilizing the transfer function, H(s), carries several limitations, which underscore the advantages of a proper transfer function model for MRI gradient systems.

### Direct insight into system stability and transient behavior

The most significant advantage of the transfer function lies in its direct exposition of system poles and zeros in the complex s-plane. The location of these poles explicitly describes the gradient system’s stability and the nature of its transient response [13, 14, 18]. For instance, whether the gradient trajectory will overshoot, oscillate, or settle after an input change is immediately apparent from the pole locations. While the frequency response implicitly contains this information, extracting stability margins or quantifying transient characteristics (e.g., damping ratios or natural frequencies) from a Bode plot alone is indirect and less robust than direct pole-zero analysis. A comprehensive transfer function model allows for clearer diagnostics of potential instabilities or undesirable ringing modes inherent to a given gradient system.

### Generality for arbitrary waveform prediction and advanced control design

The transfer function can be used as a tool for predicting the system‘s output for any arbitrary input waveform. By transforming the desired gradient waveform into the Laplace domain X(s) and multiplying by the system‘s H(s), the predicted output Y(s) can be obtained, which can then be inverse transformed to get y(t). This is fundamental for sophisticated gradient pre-emphasis schemes and real-time control [15]. While convolution with the GIRF in the time domain also achieves this, the algebraic nature of H(s) simplifies advanced controller synthesis and optimization techniques that allow to operate directly on the transfer function model.

### System interconnection and troubleshooting

MRI gradient systems are complex entities comprising amplifiers, cooling systems, coils, and cryostat eddy current paths. Understanding how these individual components contribute to the overall system behavior is critical for optimization and troubleshooting. Both frequency responses and transfer functions can, in principle, be combined to predict the behavior of interconnected subsystems. However, the algebraic properties of the transfer function, e.g., multiplication for cascaded elements and summation for parallel paths, provide a more compact and systematic framework for modeling such interconnections [13, 15]. This not only clarifies how hardware modifications affect the overall system; however, it also helps to localize the source of deviations between the predicted and measured responses, thereby supporting targeted troubleshooting. In other fields, this allows engineers to analyze how specific hardware modifications might affect the overall system transfer function, aiding in systematic design and fault diagnosis, rather than relying solely on empirical frequency response measurements of the entire integrated system.

### Model-based design and optimization

One of the primary goals of system characterization in engineering is to obtain a compact analytical model suitable for design and optimization. The transfer function, as a rational polynomial, provides this analytical form suitable for parametric system identification. This allows for the integration of gradient system dynamics into larger control system design frameworks, including the development of advanced pre-emphasis algorithms that are tailored to the identified pole-zero structure. Furthermore, a transfer function model paves the way for implementing advanced strategies such as internal model control (IMC) routines [23, 24] and observer-based control design [25, 26], which can significantly enhance system robustness and performance beyond current capabilities. Such model-based approaches can lead to more robust and higher-fidelity gradient control than purely empirical methods, potentially further alleviating the current compensation approaches.

### Beyond linear and time invariance

While the LTI assumption is the most common and has proven useful, some studies have reported gradient system behavior beyond linearity and time invariance on their MRI systems [27–29], whereas other systems appear to respond mainly linearly to temperature changes [7]. Nevertheless, if a nonlinear case is investigated, such effects should be described with frameworks beyond transfer functions, since these are strictly defined for LTI systems. From a control engineering perspective, nonlinear or time-variant dynamics would typically be described using nonlinear state-space models [30, 31]. This would allow for a model with multiple input parameters.

## Conclusion

By adopting the described terminology, the MRI community can rely on established theory from control systems and signal processing. Thus, we propose to use the following terminology: the measured impulse response in the time domain is referred to as the gradient impulse response function (GIRF). The fast Fourier transform of the gradient impulse response in the frequency domain is called gradient system frequency response (GSFR), as it indicates the corresponding attenuation/damping of the individual frequency components of the gradient system. Finally, the term gradient system transfer function (GSTF) is used for the complete mathematical characterization in the complex frequency domain, expressed as a rational polynomial representation of an LTI system. This will help to reduce existing terminological confusion while providing additional analytical tools for gradient system characterization, ultimately supporting advances in gradient system design. GIRF measurements and GIRF-based trajectory predictions have already proven to be valuable in practice and will likely continue to play an important role due to their directness and simplicity. At the same time, treating the GIRF-based frequency response as input for a subsequent, more comprehensive parametric system identification of the gradient system transfer function (GSTF) may open up further possibilities that go beyond what non-parametric characterization alone can provide. In this sense, different modeling approaches, ranging from exponentially decaying eddy current models to non-parametric LTI characterization and parametric transfer function identification, should be regarded as complementary tools, each useful for different applications and levels of complexity.

## Appendix:

### A) Determining the transfer function from the frequency response

The transient response method yields a system‘s frequency response, H(jω), across a range of frequencies, derived from the ratio of Fourier transforms of the output and input $H(j\omega )=Y(j\omega )/X(j\omega )$. The subsequent task involves converting this measured data (a set of complex numbers representing gain and phase) into a parametric transfer function H(s), which is an approximated ratio of polynomials in the Laplace domain. This process is known as parametric system identification in the frequency domain, essentially a curve-fitting problem aimed at finding the coefficients of a rational function that best approximates the measured frequency response data.

## Preprocessing measured frequency response data

Before the fitting process, typically the following preprocessing steps are performed with the H(jω) data:

- Complex form: Ensure the frequency response data is in complex form, whether as real and imaginary parts or as convertible magnitude and phase components.
- Frequency range: Focus on the frequency range where the input signal exhibited sufficient power and the system displayed significant dynamics. Avoid regions with very low signal-to-noise ratio (SNR), such as frequencies beyond the input pulse’s bandwidth or where the system’s gain is minimal.
- Averaging: If multiple transient pulses were used and their time-domain responses averaged, the H(jω) data should already be smoother. If not, consider averaging the frequency response data if multiple experimental runs were performed.
- Outlier removal: Eliminate any clear outlier data points that may stem from measurement errors.
- Weighting: Assign weights to different frequency points, for instance, giving more significance to frequencies with higher SNR or where the system’s behavior is most critical for the application.

## Choosing a model structure (order estimation)

This step is often the most challenging, requiring prior knowledge of the system. The goal is to estimate the order of the transfer function.

- Parameters to estimate:
  o Number of poles (*n*): This determines the order of the denominator polynomial in H(s) and directly influences the system’s stability and dominant transient behaviors.
  o Number of zeros (m): This defines the order of the numerator polynomial in H(s), impacting the phase response and potentially introducing notches or specific frequency-dependent characteristics.
  o Time delay: If a significant transport delay exists in the system, it is typically modeled as ${{e}^{-s{T}_{d}}}$, where ${T}_{d}$ is the delay. This term must be identified separately or incorporated into the fitting process.
- Initial guesses: Bode plot inspection (graphical method): Plotting the measured magnitude and phase response provides crucial insights:
  o Slope of magnitude plot: At high frequencies, the slope of the magnitude plot often indicates the difference between the number of poles and zeros. A slope of –20 dB/decade suggests one more pole than zero, while –40 dB/decade implies two more poles than zeros.
  o Phase shift: Each pole contributes approximately –90 degrees of phase lag, and each zero contributes approximately + 90 degrees of phase lead at high frequencies. The total phase shift can hint at the difference between the number of poles and zeros.
  o Resonances/notches: Peaks or dips in the magnitude plot signify resonant frequencies or anti-resonant (notch) frequencies, respectively, often indicating the presence of complex conjugate pole or zero pairs.
