# Correction: Naming convention for gradient system transfer function and gradient system frequency response for magnetic resonance imaging encoding field characterization

**DOI:** 10.1007/s10334-026-01336-9

**Published:** 2026-03-26

**Authors:** Niklas Wehkamp, Patrick Hucker, Johannes Fischer, Andreas Greiner, Jon-Fredrik Nielsen, Maxim Zaitsev, Robert Dehnert

**Affiliations:** 1 Division of Medical Physics, Department of Diagnostic and Interventional Radiology, University Medical Center Freiburg, Faculty of Medicine, University of Freiburg, Freiburg, Germany; 2 Laboratory for Simulation, Department of Microsystems Engineering (IMTEK), University of Freiburg, Freiburg, Germany; 3 Department of Biomedical Engineering, University of Michigan, Ann Arbor, Michigan USA; 4 Institute of Automatic Control, School of Electrical, Information and Media Engineering, University of Wuppertal, Wuppertal, Germany

**Correction: Magnetic Resonance Materials in Physics, Biology and Medicine** 10.1007/s10334-025-01314-7

In the original version of this article, title was incorrectly given as “Comment: naming convention for gradient system transfer function and gradient system frequency response for magnetic resonance imaging encoding field characterization” but should have been “Naming convention for gradient system transfer function and gradient system frequency response for magnetic resonance imaging encoding field characterization”.

